# Evaluation of Wear Behaviour in Metallic Binders Employed in Diamond Tools for Cutting Stone

**DOI:** 10.3390/ma14143988

**Published:** 2021-07-16

**Authors:** Fátima Ternero, Pedro M. Amaral, Jorge Cruz Fernandes, Luís Guerra Rosa

**Affiliations:** 1 Engineering of Advanced Materials Group, Higher Technical School of Engineering, University of Sevilla, Avda. de los Descubrimientos s/n, 41092 Sevilla, Spain; fternero@us.es; 2 IDMEC—Instituto de Engenharia Mecânica, Instituto Superior Técnico, University of Lisboa, Av. Rovisco Pais, 1049-001 Lisboa, Portugal; Pedro.Amaral@tecnico.ulisboa.pt (P.M.A.); cruz.fernandes@tecnico.ulisboa.pt (J.C.F.)

**Keywords:** metal-bonded diamond tools, binder wear behavior, metallic binders, stone machining

## Abstract

A type of disc-on-plate test methodology was used to determine the wear behavior of metallic binders employed in the manufacturing of diamond impregnated tools. The disc consists of a special circular wheel that allows the binder materials alone (i.e., without diamond, but sintered under conditions identical to those of the complete tool) to be tested against a plate of stone material under pre-determined testing conditions. The testing conditions are intended to be equivalent to those used in the industrial processes. Using plates of five types of granite and one type of marble, this work comprises wear tests of 15 different types of metallic binders and two sintering modes conducted under, at least, three different values of contact-force. The analysis of the results demonstrated that the wear of the binders can be related to their mechanical properties through an empirical expression. The larger the difference between the characteristics of the tribological pair (binder versus stone), the higher is the correlation between the experimental wear data and the values given by the empirical expression. The relationships presented in this work allow predicting the wear behavior of the binder, and therefore may help in the design process of diamond tools. There was a clear difference between the wear behavior of metallic binders when they were employed against the two main classes of stone under analysis (marble and granite).

## 1. Introduction

The act of cutting a stone may be defined as a process involving the removal of a pre-defined quantity of material carried out by a particle in motion (normally defined as the abrasive particle). The process usually employs very hard granular particles, such as diamond, and each particle plays a critical role by driving the breakage of a, usually small, amount of stone material after being forced into the surface of the workpiece.

In general, we may divide the several operations dealing with stone cutting into the production processes that are usually part of a transformation activity. To become a product, the raw material needs to be transformed through a variety of these processes. Usually, the first (primary) transformation processes are employed to slab a block coming from a quarry into smaller volumes that are cut into consistent sections (slabs). Secondary transformation processes normally deal with these large slab sections obtained from the blocks.

These primary and secondary transformation processes have led to an increase in the use of metal-bonded (sintered) diamond tools, not only for cutting stone, but also for cutting other hard materials, such as glass and concrete. Today, diamond tools are employed in almost every process of the transformation activity, and therefore, these developments have also led to an increase in research dealing with optimum tool design. For example, there have been studies dedicated to the design of brazed diamond grids: from the earlier work of Sung [1] to recent review work by Long et al. [2]; models and theoretical analyses of stone sawing with diamonds [3,4,5,6,7], with an emphasis on diamond impregnated segments [7,8,9]; industry-oriented evaluation of diamond tools [10,11,12], including their long-term performance [13]; studies on the diamond retention capacity of metal bond matrices [14]; and optimization of the sintering process of the binding phase and the diamond composite materials [15,16,17,18].

Research studies are of particular interest for setting up the conditions for which diamond tools may (or may not) be employed in specific applications. This work gives an insight into one of these studies by analyzing the application of different metallic powders, applied as binding materials in the production of powder sintered tools containing diamond grit for cutting stones. The empirical knowledge observed in current applications creates the need to better characterize the behavior of such matrix materials and to evaluate their relationship with the tool performance. If the former objective is possible to achieve by developing and validating new characterization methods (such as those in the present work), the latter sets one of the most difficult activities in this research field, due to the great complexity involved in the cutting process; it involves different diamond tool products normally designed to fit a certain application, different types of stone materials, and different technological solutions used with certain types of equipment in order to obtain the final stone product.

In general, in order to simplify the analysis, theoretical studies tend to consider “average” stable conditions during a cutting process. Hence, we may define that, when a single abrasive particle removes a small amount of stone, the force resulting from the penetration and consequent breakage of material will generate a stress field in the binder. The stress field will vary over time, while the quantity of material removed is decided by the cutting conditions set up initially. As would be expected, there will be a relationship between the volume removed by the particle and the resulting force at each instant [6,19].

It is important to emphasize that this event is cyclic and normally occurs at a fast rate. The (cyclic) stresses need to be addressed when optimizing the properties of the binders. This effect is even more important if the process involves alternate motion, i.e., when the particle removes material in two alternate directions. This is usually the case when blade (gang) sawing processes are employed for slabbing stone blocks. In summary, depending on the stress fields occurring in the binder (derived from the cutting conditions and type of material being removed) different diamond retention capacities are observed [14].

During cutting, the amounts of material being removed (normally mixed with the lubricant and named swarf) need to be withdrawn from the location where the abrasive interacts with the stone. In normal cutting conditions, the swarf will also interfere with the binder when contacting its surface. Depending on the working conditions and the physical characteristics of the type of material being cut, this contact will play a critical role in the wear resistance of the binder.

An adequate level of diamond particle retention will be achieved when a reasonable number of diamonds at the surface are active, i.e., performing an effective cut (originating an expected force), and held in the binder long enough to yield an efficient process (envisaging, for example, a lower cost by increasing the tool durability).

For achieving the proposed cutting outcome, the role of a binder may be analyzed in terms of the abovementioned interactions and quantified by function of its intrinsic characteristics, which can be related to two effects:

- Diamond Holding Capacity: an attribute of the binder related to the capacity for resisting the continuous impacts/forces/stresses caused by the penetration of the diamond into the stone during cutting, with the objective of avoiding a premature loosening of the particle (i.e., losing a particle that has not reached the end of its expected working life). In general, the diamond holding capacity will be higher the better the mechanical tensile strength conditions in the binder in which the particles are embedded and the higher the boundary adhesion between the binder and the particles.
- Wear resistance: an attribute of the binder, related to its mechanical properties, and that depends on the wear mechanism originated by the cutting process and the class of stone. The relationship between wear resistance and binder properties will differ from case to case (from severe erosive wear mechanisms to adhesive wear).

**Figure 1:**
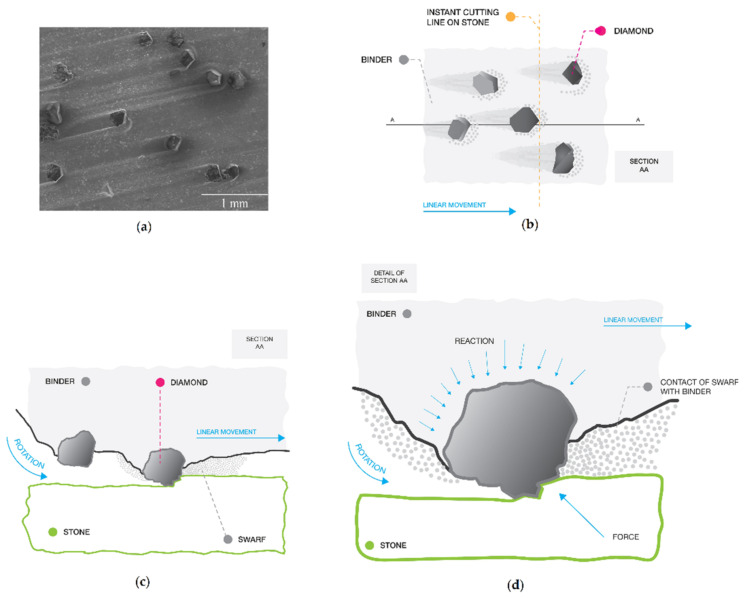
Schematic representation of the interaction between an abrasive particle in action (e.g., diamond) embedded in a binder and the material (e.g., stone) being cut; (**a**) optical observation of the top section of a diamond segment; (**b**) schematic view of the top section of a segment, representing a location where an instant cut is taking place on the stone; (**c**) section AA; (**d**) detail showing the locations where the binder is affected.

Taking into account these two parallel effects it is possible to clarify how they interact during a standard process. The schematic representation depicted in Figure 1 shows how the phenomena associated with stone cutting is typically represented in the vicinity of each active abrasive particle (usually diamond). Figure 1a is an optical observation of the top section of a diamond segment showing a “tail” texture in the binder due to the cutting; and, it should be noted, the “tail” helps support the diamond. Figure 1b is a schematic view of the top section of a segment, representing a location where an instant cut is taking place on the stone. Figure 1c represents section AA, showing a diamond initiating penetration into the stone, as well as the swarf originated by the cutting. Finally, Figure 1d is a detail of Figure 1c showing the locations where the binder is affected, namely, by the wear due to permanent contact with the swarf and reaction forces generated during the diamond penetration.

From the foregoing considerations it can be understood that the cutting process of stone materials is a complex topic of research due to the very high number of variables affecting the outcome. Theoretical approaches may explain the phenomena, but they are not fully adequate when industrial optimization dealing with diamond tools is required (e.g., developing a new diamond tool product, optimizing a specific cutting process with respect to a given stone material). Therefore, as in many other situations, the most rigorous evaluation of the cutting behavior is achieved when test conditions are defined in line with those occurring in a real environment. This implicates the development of adequate test methodologies aiming at an accurate characterization of diamond tools, binders, diamonds, and stones.

Systematic research studies conducted with IST-Lisbon classification equipment (made by IST, Lisbon, Portugal) [10,13,17] have shown a relation between the wear performance and other characteristics of the tools; for example, a relationship between the tool wear rate (tool weight-loss per run) and the mechanical parameters of the different metallic binder materials used to manufacture the correspondent tools [20], or a relationship between energy consumption and the magnitude of forces acting between the tool and the workpiece when different working conditions are used [10].

On the assumption that the wear of the binder is thought to play an important role in the overall tool wear performance, one may conclude that binder materials need to be properly characterized, mainly from two perspectives:

(i) A mechanical characterization, to establish first selection criteria when designing new tools, especially when it is possible to compare similar processes using equivalent tools with known binder properties. This characterization follows test methodologies that are well established [21].
(ii) A wear characterization, where the binders are tested in comparable situations from those observed in real cutting conditions. For assessing relevant wear parameters related to the cutting processes, to date no works have been observed in the open literature.

This paper describes a test methodology for determining binder wear properties in order to make a comprehensive evaluation of wear behavior in metallic binders employed in diamond tools for cutting stone. The technological conditions employed aim at the evaluation of the wear in the binder caused by testing it against different types of stones. Hence, one valid condition for conducting the test is that the selected stones must not show significant wear when compared to that suffered by the binder. This condition is evaluated in detail during this work.

The experimental work comprises the wear characterization of 15 different metallic binder materials, evaluated against six different types of stone materials. In order to evaluate binders produced for different cutting applications, the materials tested in this work were manufactured using two different sintering conditions normally defined in the tool sector: (i) hot pressing; and (ii) free sintering.

While the main objective of this work was to evaluate the wear in the binders using working conditions close to reality, a secondary but no less important objective was to define the test methodology with relation to the type of tool in which the binder is normally employed: segmented (hot-pressed binders) or continuous (free-sintered binders).

## 2. Materials and Methods

### 2.1. Powder Sintered Binders and Their Properties

**Figure 2:**
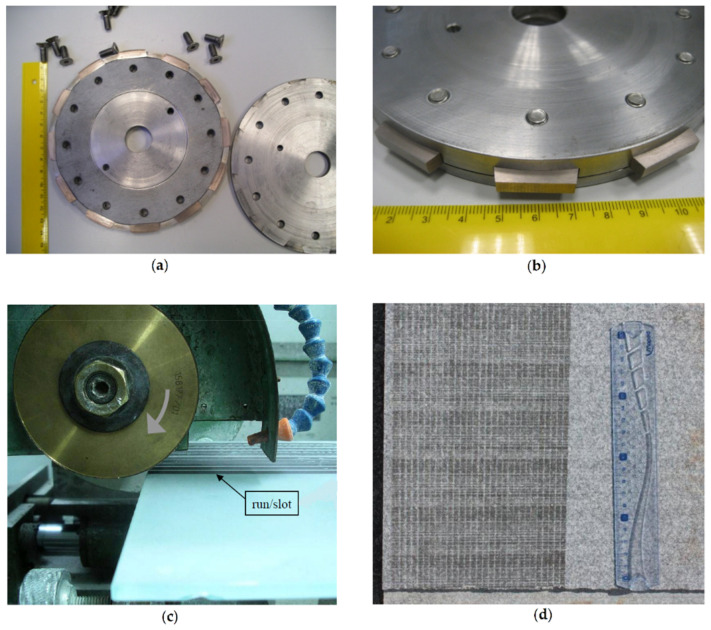
(**a**) General view of the rig used to test the binders produced as segments by hot pressing; (**b**) detail of the system used to fix the binder specimens; (**c**) general view of the continuous wheel used to test the binders produced by free sintering; (**d**) a set of runs obtained during a wear test in a granite plate (tile), showing the marks left after a series of runs.

In the present work, traditional diamond tools are replaced by special circular wheels that allow the binder materials (without diamond) to be tested against stone materials under pre-determined testing conditions. Since the binders alone do not cut the stone, the tests are made by setting a pre-determined force against the stone material used. As in the analysis of the tools, the wear in the binders is assessed after a pre-determined (constant) number of runs. Taking into account that binders have different densities, the loss of volume is calculated from the measurement of the loss of weight. Figure 2 shows the special circular wheels designed for assessing binder wear rate, as well as an example of a stone surface after performing several tests. It should be noticed that, to perform tests in binders produced as segments, a special test rig was designed and manufactured. Preliminary tests results using this particular system have been presented elsewhere [21].

In this work, thirteen different metal-powder compositions, normally employed in industry, were selected from a wide range of available powders. All the powder mixtures were produced by Umicore/Eurotungstene (Brussels, Belgium). Note that, from the thirteen different metal-powder compositions, a total number of 15 different types of binders were obtained through sintering (see Table 1). In order to demonstrate how sintering conditions significantly affect the wear behavior of the consolidated body (the binder), two alternative sintering conditions were used. Hot pressing is traditionally described as the main sintering technology to produce diamond segments for sawing tools. While, free sintering is a technology employed for producing diamond tools used in milling processes, mainly when complex geometric attributes are required or, in the case of sawing, to produce continuous tools (i.e., not segmented). The term “free sintering” is normally defined in the tool sector when the consolidation is made without applying the heating and pressure simultaneously. Usually, the mold employed when using this technology is made of special high-temperature-resistant alloy steel.

From the total of 15 different types of binders obtained through sintering, nine were obtained by hot pressing (HP). The tests performed in the binders produced as segments (HP) were all conducted using the same test rig, as depicted in Figure 1a. The binders obtained by free sintering (FS) were consolidated directly in the metal supporting structure (made of brass) and this implicated that six different wheels had to be manufactured in order to test each type of binder.

The production of the HP binders followed the same production procedure for manufacturing the diamond segments used in cutting tools: after the formation of a green body by uniaxial pressing of the mixed powders, the bodies were sintered at temperatures ranging from 750 to 900 °C, with a pressure within 33–35 MPa, for a period of 3 min.

The manufacturing process of the FS binders comprised: (i) formation of a green body using a similar procedure to HP; (ii) a free sintering cycle during a pre-defined period of time (from 20 to 40 min), with plateau temperature in the range of 700 to 850 °C; (iii) a uniaxial pressure (up to 35 MPa) applied to the body immediately after it leaves the furnace in order to achieve final densification. After final pressure, the body is cooled in air, at room temperature.

To evaluate the mechanical properties of the binders, additional test specimens of each binder were also manufactured from the same batch. The characterization of the metallic matrices comprised the following methodologies:

- The density of the binders (ρ) was determined by the Archimedes/water immersion method using an Electronic Densimeter EW-200SG (Alfa Mirage Co., Ltd., Osaka, Japan).
- Vickers hardness (HV) was determined using the hardness tester MITUTOYO AVK-C2 (Mitutoyo Corporation, Kanagawa, Japan) with a load of 1 kgf.
- Determination of the dynamic Young’s modulus (E) was carried out using RFDA equipment (made by IMCE, Genk, Belgium) in flexural vibration mode [11].
- Other mechanical properties: rupture stress (σ) and toughness modulus (m––obtained from the area bellow the stress/strain curve) were evaluated through tensile tests conducted on cylindrical specimens with a reduced-section length of 30 mm and 4 mm in diameter, using an Instron servo-hydraulic machine Model 8502 (Instron, Norwood, MA, USA) under a constant crosshead travelling speed of 0.5 mm/min. The test procedure employed is described in more detail in [11].

The chemical elements present in each powder composition, and the density and mechanical properties of the 15 different types of binders, are presented in Table 1.

### 2.2. Stone Materials

To better evaluate the binder wear behavior, five types of commercial granite (“Porriño”, “South African Black”, “SPI-Azul Alpalhão”, “Rosa Monção”, and “Cinza Pinhel”) were used during the study (see brief description in Table 2). To evaluate the test method using a “softer” stone material, tests were also conducted on a Portuguese marble “Mármore Estremoz”. In general, the rocks were selected to establish a comparison with a medium hardness class of granite, known worldwide: the “Porriño”.

### 2.3. Wear Equipment and Test Procedure

**Figure 3:**
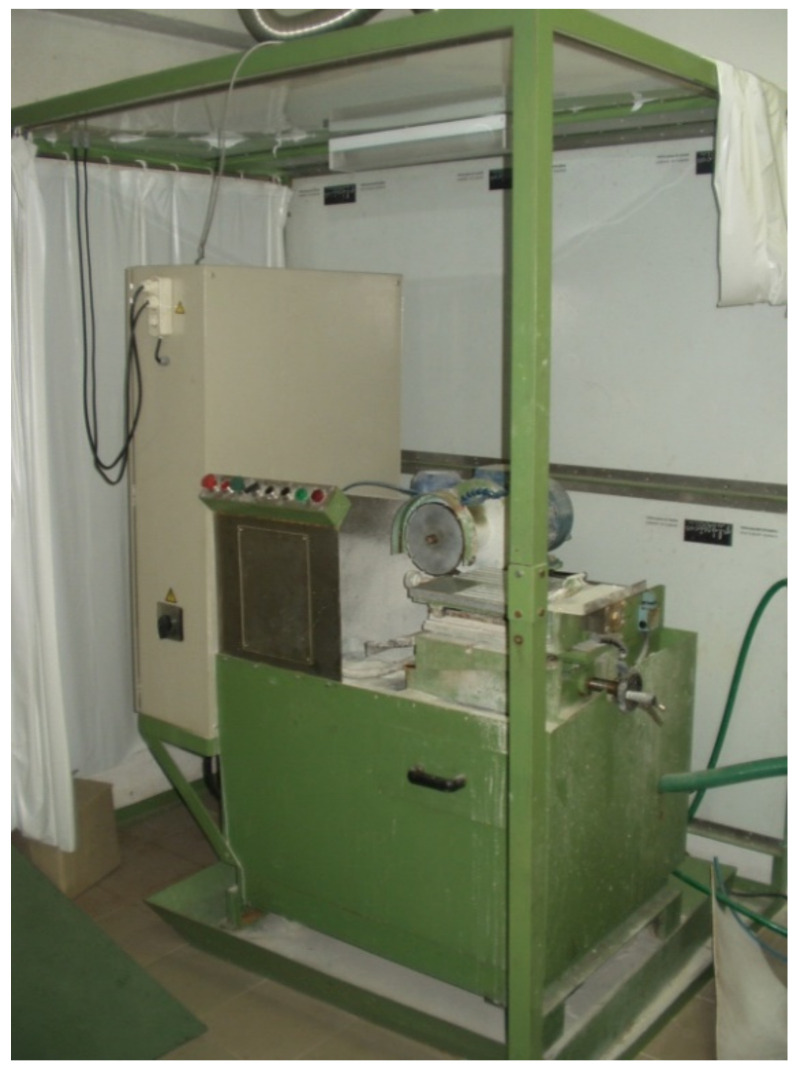
Test Equipment: the I.S.T-Lisbon Classification Machine.

The wear tests for characterizing the behavior of the binders were conducted with the “IST-Lisbon classification machine”: a prototype, specially designed and manufactured for applied research studies. When testing diamond tools, this equipment allows the use of circular discs (showing either a segmented or continuous cutting element) with diameters between 100 and 400 mm. The wear tests can be conducted against several types of workpiece materials. The tools are coupled to an electric motor capable of supplying a constant rotational speed (between 500 and 2900 rpm). Another motor moves the tool forward (at a constant infeed speed, ranging from 5 to 25 mm/s) towards a platform, to which the workpiece material is clamped. The equipment allows independent control of the following parameters: rotational speed, infeed speed, depth of cut, applied forces, and flow of coolant (liquid lubricant). The equipment also allows real-time acquisition of the following output parameters: vertical force, horizontal force, electrical energy spent during operation, and vibrations of the rotor shaft. More details of the present version of the test apparatus have already been presented elsewhere [10,20]. Figure 3 shows a general view of the equipment used in the present work.

The testing procedure establishes a certain number of cutting slots or runs. The objective is to determine the tool weight loss monitoring the resultant forces (generated by the contact between the binder and the workpiece material). The total number of runs depends on the characteristics of the workpiece material and type of tool.

The test procedure for evaluating the wear behavior of the binders is relatively simple: the stone workpieces (tiles) have standardized surface planar dimensions of 300 × 300 mm^2^. It is important to have a very planar surface since it is essential to create, from the beginning of the test, a stable contact condition with the wheel. Taking into account the nature of the contact, it is also important that each stone surface possesses a constant roughness. Consequently, the testing methodology can be summarized in the following steps:

1: The wheel (where the binder material is set) is clamped into the test equipment.
2: The stone is positioned on the worktable (equipped with load cells) and clamped so that vibrations do not affect the conditions for measuring the components of the forces.
3: The input parameters (working conditions) are introduced into the software according to a previously set design of experiments.
4: A pre-determined vertical force is established for the contact between the wheel (binder) and the stone material. This force is obtained by pushing the wheel, in a static condition, against the stone plate and monitoring the force till the pre-determined value is established. The output force obtained once the test is initiated (at dynamic regime) is always slightly different for that obtained when the wheel is positioned at this stage (static positioning). Therefore, experience is necessary to relate this aspect, in order to estimate the required output.
5: All counters are zeroed before starting a test.
6: During the test, the output parameters are shown in the software and stored according to a pre-determined acquisition frequency (around 10 Hz). These parameters are the vertical and horizontal forces and the electric energy consumption. Since, for the conditions used in this work, the forces and the energy are related, only force is used for analyzing the results.
7: Every time a new wheel with a new binder is installed, some runs are made till the system stabilizes the forces (within a given margin of error). This normally occurs after 1 or 2 runs in a continuous wheel (used to test the binders produced by free sintering), but a larger number of runs may be needed in a wheel with binder segments, since the installation of new segments in the rig normally fails to accomplish a perfectly circular shape.
8: The counters acquire data till the run is performed and the wheel moves forward at a nearly constant force.
9: To analyze the repeatability, at least 4 runs are performed for each of the working parameters defined in 3. More test runs may be needed if the total loss of weight in the binder specimens is lower than 0.5 g. This condition is critical when the wear tests are made against stones that do not cause significant weight loss in the binders (e.g., marbles).
10: The forces acquired produce a lot of data that are treated after ending all test sequences. From the output data it is possible to calculate the arithmetic mean value of the forces and the corresponding standard deviation.
11: To analyze the reproducibility, the test procedure is repeated in three separate rounds of experiments (from item 1 to 10).
12: This test sequence is repeated for each working condition (from item 1 to 11). Working conditions are defined according to the design of experiments, which may vary depending on the objective of the study. In the present study, the results of wear (total loss of volume calculated from the loss of weight) were determined for, at least, 3 different forces that are intended to simulate different contact conditions between the binder and stone material, such as those occurring when the binders (with diamond embedded) are performing real cuts.

The testing conditions were defined in line with previous studies performed in binders employed in continuous tools [22,23]. Although the infeed speed (*v* = 25 mm/s), rotational speed (rot = 2900 rpm), and lubricant conditions (water flow = 1 L/min) were kept constant, the derived peripheral speed differs depending on the type of wheel being tested. Table 3 outlines the test conditions along with the relevant geometric details of both wheels.

Finally, experimental results derived from these tests are represented by plots showing the arithmetic mean value of the wear measured as tool volume loss per run (V) versus resultant force (F, obtained from the vertical and horizontal components of the force measured by the load-cell table).

## 3. Results 

**Figure 4:**
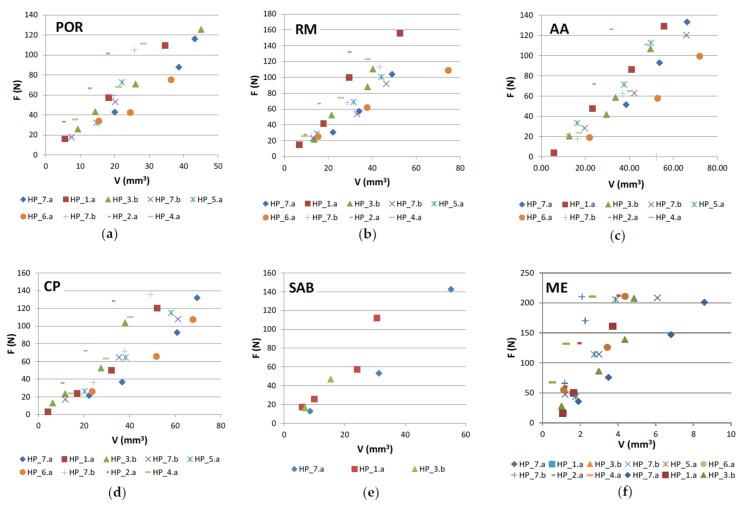
Graphical representation (wear plot) of the overall results obtained during this work using the test rig for binders produced as segments. The results were obtained in 6 different stone materials, including the marble (ME). (**a**) in “Porriño” (POR) granite; (**b**) in “Rosa Monção” (RM) granite; (**c**) in “Azul Alpalhão” (AA) granite; (**d**) in “Cinza Pinhel” (CP) granite; (**e**) in South African Black (SAB) gabbro; (**f**) in “Mármore Estremoz” (ME) marble.

**Figure 5:**
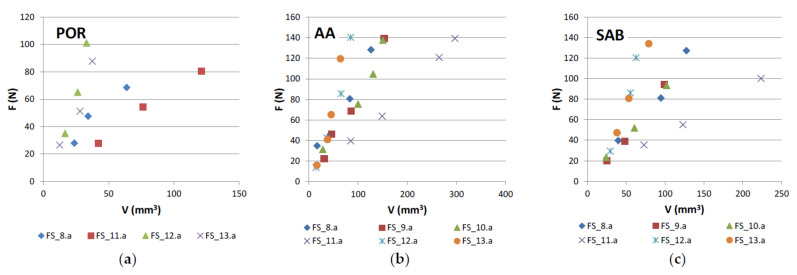
Graphical representation (wear plot) of the overall results obtained during this work using the wheel, where a continuous layer of binder was sintered. The results were obtained using 3 different stone materials. (**a**) in “Porriño” (POR) granite; (**b**) in “Azul Alpalhão” (AA) granite; (**c**) in South African Black (SAB) gabbro.

The following graphical representations (wear plots) show the overall results obtained during this work, either using the test rig for binders produced as segments (Figure 4) or using the wheel, where a continuous layer of binder was sintered (Figure 5). As may be observed from the plots, all cases show a great linearity between (F) and (V). This demonstrates that the test methodology is, not only useful to evaluate the wear in the binder caused by each stone material, but also that it is particularly sensitive to wear variation when different contact conditions are established. The later outcome is helpful for establishing criteria towards binder selection, because it allows reproducing, as much as possible, real cutting conditions.

**Figure 6:**
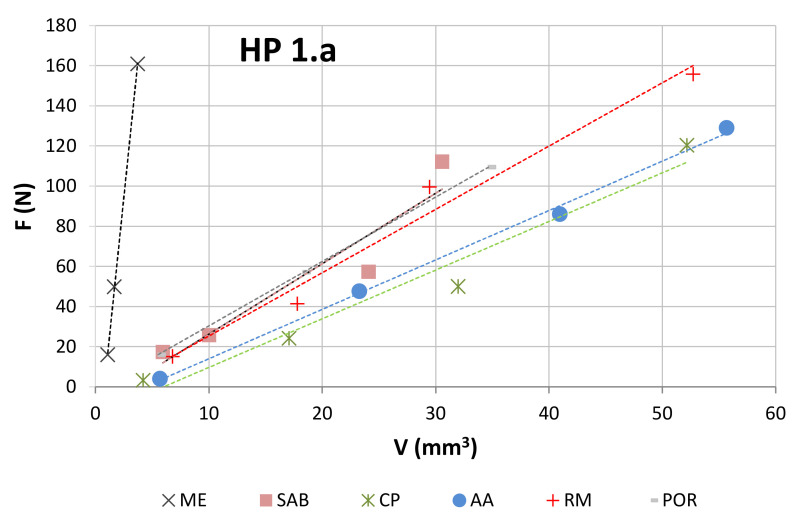
Graphical representation (wear plot) of the results obtained when testing the HP_1.a binder against 6 types of stone.

The evaluations of the repeatability and reproducibility were performed by evaluating the variations in V and F during the test. Repeatability was assessed by looking at the standard deviations of V and F after performing a minimum of four runs for each working condition. Reproducibility was assessed by calculating the standard deviation of the arithmetic mean values of V and F after performing a minimum of three rounds. In general, the variation of V was found to be less than 9%, and the variation of F was less than 6%. As an example, Figure 6 shows how the HP_1.a binder behaved when tested against different stone materials. The details of the test results obtained for HP_1.a binder are presented in Table 4. In this case, four different conditions (of contact-force) were used with RM, AA, CP, and SAB; and only three different conditions (of contact-force) were used with POR and ME.

As it is possible to observe from Figure 6, there are two main regions defined in the wear plot. One is related to the test conducted on the marble (ME) and the other corresponds to the tests made in the granite stones. This indicates that the behavior of each individual binder was not significantly affected by the type of granite used, but it was strongly affected when a totally different class of stone (marble) was used. This issue will be discussed further subsequently.

## 4. Discussion

Previous investigations [12,23] have shown that the wear of binders can be related with their mechanical properties through an empirical expression with the following proportionality:

(1) $$V=\alpha_{1}\cdot F^{a}\cdot A$$

with

(2) $$A=\frac{1}{{\mathrm{HV}}^{b}\;E^{c}\;\sigma^{d}\;m^{e}}$$

where *α*_1_ is a constant that depends on the test setup, and *a*, *b*, *c*, *d,* and *e*, are constant exponents that are determined by fitting Equation (1) to the experimentally measured data [12]. Taking into account the large number of metallic binders and stones used in the present work, the evaluation took into consideration the use of: *a* = 1.0, *b* = 0.5, *c* = 1.5, *d* = 0.5, and *e* = 0.05. These values attributed to *a*, *b*, *c*, *d,* and *e*, are considered suitable for testing binders, either using sintered segments or continuous wheels, against any type of stone material. This assumption is also discussed in the following.

**Figure 7:**
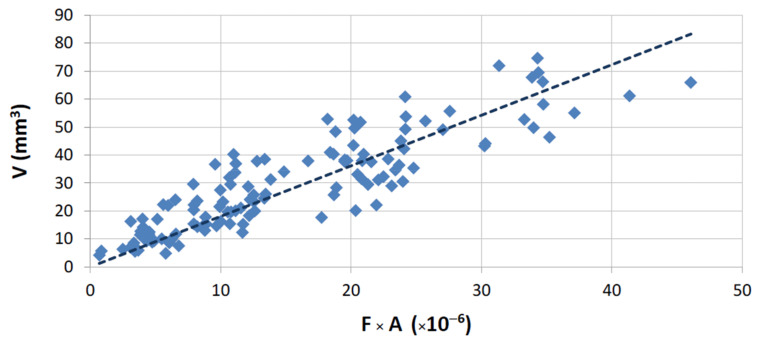
Graphical representation of Equation (1); correlation for results in HP binders tested against the commercial granites (R = 86.2%; *α*_1_ = 1.81 × 10^6^).

**Figure 8:**
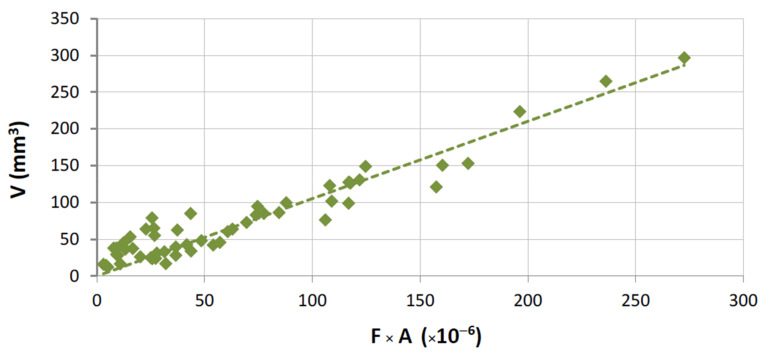
Graphical representation of Equation (1); correlation for results in FS binders tested against the commercial granites (R = 94.1%; *α*_1_ = 1.05 × 10^6^).

**Figure 9:**
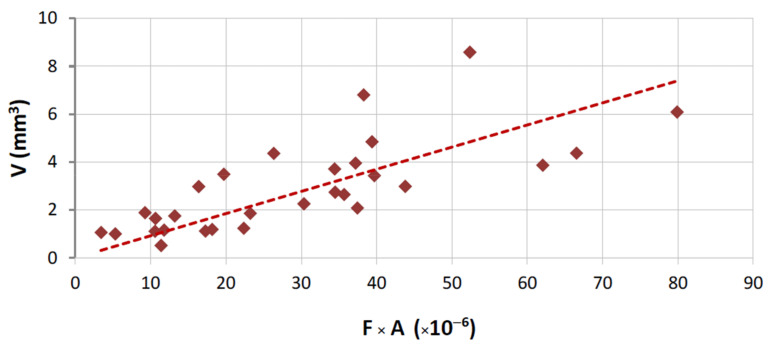
Graphical representation of Equation (1); correlation for results in HP binders tested against the marble (R = 70.4%; *α*_1_ = 0.09 × 10^6^).

A graphical representation of Equation (1) is presented in Figure 7 for the overall results obtained from tests with HP binders. The same equation is plotted in Figure 8 for the wear tests performed with the FS binders. In both plots, only results derived from tests performed against commercial granites are shown. For the case of marble, the application of Equation (1) is depicted in Figure 9.

As can be clearly observed from Figure 9, Equation (1) does not seem totally suitable to evaluate the binder wear behavior against marble. This particular issue can be explained by the fact that the harder binders (hot pressed) were tested against a much softer surface (when compared to the remaining stone materials). The results indicate that the tribological pair (harder HP binders versus marble) may not fulfil one important requirement of the present test methodology: the marble is also being worn during the contact with the binder, modifying the type of contact, and hence the inherent wear mechanism. This explanation is also supported by observing the behavior of a single binder when tested against both classes of stone materials (a marble and several granite stones); see the wear plot in Figure 6.

Concerning the remaining plots of Equation (1), the best fitting seems to be observed when FS binders were employed. Similar reasons from those outlined with the marble may help to explain the difference in the results between the FS and HP binders. Harder binders produce a more forceful contact with the stone surface, causing slightly different wear conditions; i.e., the harder binders tended to cause wear even in the granite stones.

As was discussed when presenting Table 2, the conditions during the wear tests were not totally coincident because of the geometrical differences between the two types of rig (see Figure 2). Therefore, the results shown in Figure 7, Figure 8 and Figure 9 cannot be directly compared. Taking into consideration the characteristics of both types of contact, Equation (1) has been modified in order to become “normalized”, i.e., to allow a comparison of results for any test setup. For carrying out the “normalization”, one needs to estimate the total amount of area contacting the workpiece during each run, considering the width of the contact (w), the partition (r), the rotational speed (rot), and the period of time (t) for which a given length of a single run (L) is performed. Equation (3) shows how it is possible to calculate the normalized volume loss per run (Vn):

(3) $$\mathrm{Vn}=\frac{V}{2\pi \times D\times r\times w\times \mathrm{rot}\times t}$$

On the other hand, the resultant force (F) depends on the contact interaction. To calculate the normalized force (Fn) one can divide the force by the width of the binder that is in permanent contact:

Fn = F/w(4)

This implies that Equation (1) can be represented in a normalized manner:

Vn = *α*_2_ × Fn*^a^* × A (5)

where *α*_2_ is also a constant (but different from *α*_1_).

**Figure 10:**
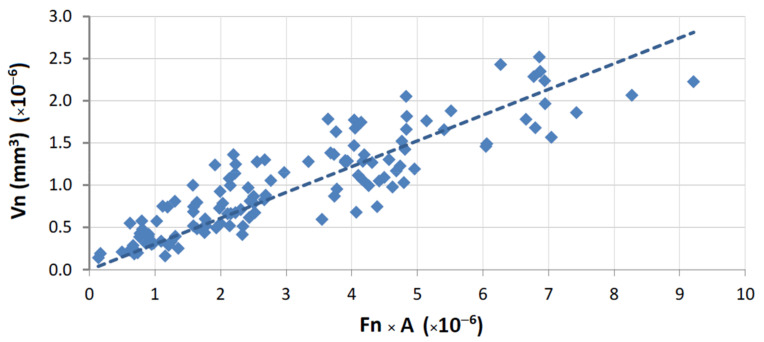
Graphical representation of Equation (5); correlation for results with HP binders tested against the commercial granites (R = 86.1%; *α*_2_ = 0.31).

**Figure 11:**
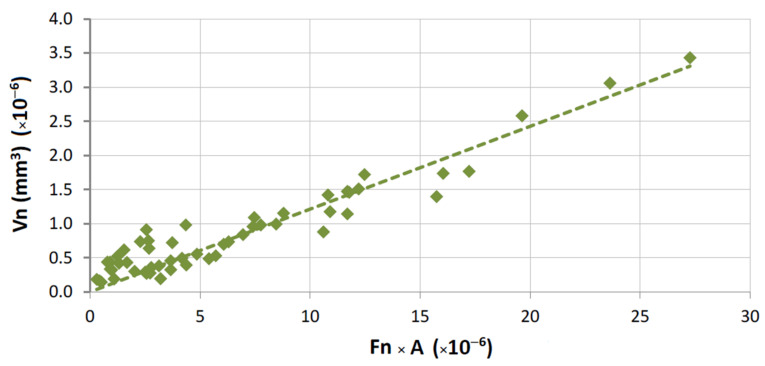
Graphical representation of Equation (5); correlation for results with FS binders tested against the commercial granites (R = 94.1%; *α*_2_ = 0.12).

**Figure 12:**
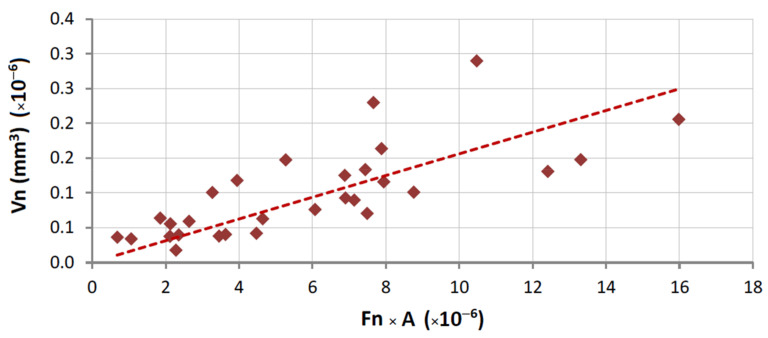
Graphical representation of Equation (5); correlation for results with HP binders tested against the marble (R = 70.4%; *α*_2_ = 0.0156).

Similarly to what was already done for the application of Equation (1), Equation (5) is graphically represented in Figure 10, Figure 11 and Figure 12, concerning the overall results obtained.

With the present results it is possible to evaluate the three situations (concerning Figure 10, Figure 11 and Figure 12) by comparing the values of *α*_2_. For each contact condition, we can expect different binder wear behavior, which seems to depend on three factors:

- The normalized force to which the binder is submitted.
- The characteristics of the contact (which depend on the type of test rig).
- The difference between the mechanical properties of the materials that constitute the tribological pair (the binder and the stone material); the greater are the differences, the clearer is the applicability of Equation (5).

The advantage of constructing “normalized” plots, like Figure 10, Figure 11 and Figure 12, is that they provide information which is independent of the test setup and dimensions of the tool.

## 5. Conclusions

In this paper, using a type of disc-on-plate arrangement, we showed the usefulness of a robust test methodology for determining the wear behavior of binders employed in the manufacturing of diamond tools. The methodology showed reliable results for determining the wear resistance of sintered bodies using a rotating wheel, where the test specimens are fixed and pressed against a standard plate (made of a stone), while moving along a pre-described distance. Among other parameters that are thought to play an important role, the determination of a normalized force/pressure parameter was found to be critical for understanding the wear behavior of the binders (volume or weight loss) using different contact conditions. It should be noted that this procedure aims to test binder wear behavior using approximately real cutting conditions, since the standard plate can be of the same material that is cut with diamond tools and employing the metallic binder that needs to be characterized. Standard plates can be of almost any kind of material used in real conditions: marble, limestone, or granite, or even made of concrete, reinforced concrete, glass, polycarbonate, or agglomerated stone.

In summary, we can conclude that:

- The use of the present test methodology showed robustness when measuring the different wear results and representing the wear plots, and independently of the type of binder used, the test configuration (continuous or segmented), the force employed, and the material against which the binders were tested.
- The results of wear when testing a single binder against different stones (Figure 6) show a clear difference between the two main classes of stones under analysis (marble and granite). This suggests a difference in the contact, related to nature of the stones. In other words, when the metallic binders are tested against the granite there is a clear difference between the characteristics of the tribological pair (granite versus metallic binder). Granites have a higher influence on the wear of the binders, and hence, even if we change slightly the nature of this class of stone (suggesting a difference in the mineralogical composition), its average hardness induces a similar effect/behavior in a single type of binder. On the contrary, when using a standard plate of marble, the results suggest that the metallic binder also induces wear in the stone. By having two materials wearing at the same time, the difficulties in relating the binder properties with the wear behavior increase significantly (see Figure 12).
- The relation proposed in both Equation (1) (when test configuration is standard) and Equation (5) (when comparing different test configurations) gives a better fitting with the experimental data when metallic binders are tested against granite stones (R = 86.1% for segmented specimens and R = 94.1% for continuous contact). The difference between these two results may derive from the fact that the binders used in continuous wheels (free sintering) are softer than those employed in segmented configurations (hot pressing). In line with the previous item discussed, the larger the difference between the characteristics of the tribological pair, the clearer seems to be the relationship between the experimental wear data and the binder’s mechanical properties.
- Observing Figure 10, Figure 11 and Figure 12, we conclude that *α*_2_ varies depending on the type of contact (42% lower when using a continuous wheel) and on the class of material (95% less if a marble is used).

The relationships obtained in this work show the applicability of the test methodology to fully characterize a binder, allowing the prediction of its wear behavior, which will help in the design process of diamond tools (in this case, for circular sawing processes). This testing methodology has also given good indications concerning its rapid use for optimizing new diamond tools that will be employed in the construction sector.

## Figures and Tables

**Table 1:** Type of binders used, showing the chemical elements present in each powder composition, density, and relevant mechanical properties (HV: Vickers hardness, E: Young’s modulus, σ: rupture stress, and m: toughness modulus).

Type of Binder ^1^	Elements in Composition	ρ (g/cm^3^)	HV (MPa)	E (GPa)	σ (MPa)	m (MPa)
HP_1.a	Co, Cu, Fe	8.08	2533	195	832.9	28.61
HP_2.a	Co	8.93	2639	215	932.8	17.34
HP_3.a	Co, Cu, Fe	7.97	2707	211	1000.5	8.79
HP_3.b	Co, Cu, Fe	7.94	2796	217	713.7	22.15
HP_4.a	Co, Cu, Fe	8.29	2744	217	989.0	9.71
HP_5.a	Co, Cu, Fe	8.26	2683	186	625.3	1.12
HP_6.a	Co, Cu, Fe, Sn	8.45	2559	175	702.8	1.57
HP_7.a	Co, Cu, Fe	8.44	2919	181	783.9	2.30
HP_7.b	Co, Cu, Fe	8.37	2840	175	468.0	0.61
FS_8.a	Cu, Sn, Co	8.01	2247	130	270.8	0.30
FS_9.a	Co, Cu, Sn	8.10	1682	110	278.9	1.65
FS_10.a	Co, Cu, Sn	8.20	1325	125	298.1	0.67
FS_11.a	Cu, Sn	8.30	1500	102	191.6	0.24
FS_12.a	Co, Cu, Fe	7.38	2674	215	422.0	0.46
FS_13.a	Fe, Cu, Co	7.40	2349	220	977.5	3.38

^1^ HP: hot pressing. FS: free sintering. a: standard sintering temperature. b: alternative sintering temperature.

**Table 2:** Brief description of the rocks used in this work.

**“Porriño” (POR)**		**South African Black (SAB)**	
Biotitic **granite**, with large grains and with some irregular fractured megacrystals of dark rose K-feldspar		**Gabbro**, with a homogeneous distribution of very dark pyroxene and grey feldspar grains (in the boundary of the former)	
Quartz (35%) K-feldspar (40%) Plagioclase (13%) Biotite (11%) Other (1%)	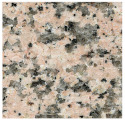	Plagioclase (55%) Pyroxene (45%)	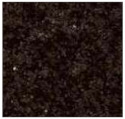
**“Azul Alpalhão” (AA)**		**“Rosa Monção” (RM)**	
Two mica **granite** (mostly biotitic), with a well distributed fraction of fine grains (light grey)		Biotitic **granite**, with large grains and with some megacrystals of light rose K-feldspar	
Quartz 30% K-feldspar (40%) Plagioclase (12%) Biotite (10%) Muscovite (7%) Other (1%)	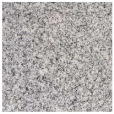	Quartz (40%) K-feldspar (40%) Plagioclase (10%) Biotite (10%)	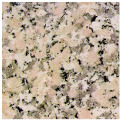
**“Cinza Pinhel” (CP)**		**“Mármore Estremoz” (ME)**	
Two mica **granite** (mostly biotitic), with medium to coarse grains and presenting a few megacrystals of feldspar		Calcitic **marble**, showing a fine to medium grained granoblastic texture	
Quartz (35%) K-feldspar (40%) Plagioclase (12%) Biotite (10%) Muscovite (2%) Other (1%)	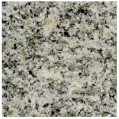	Calcite (99%) Other (1%)	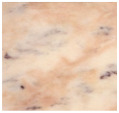

**Table 3:** Details of each type of wheel used to assess binder wear, including the derived peripheral speed.

Type of Wheel	Nominal Diameter, D (mm)	Nominal Partition (r = total Length of Binder Material in the Wheel/Perimeter)	Nominal Width of Contact, w (mm)	Distance of Each Run, L (mm) ^1^	Peripheral Speed, *p* (m/s)
Segmented	156	0.55	5	190	19.7
Continuous	125	1.00	10	190	23.0

^1^ The equipment used obliges the wheel to follow a forward and backward movement, always touching the stone plate. However, the distance of each run is determined by measuring only the length of the forward movement, during which all outputs are monitored and stored. The backward movement is not considered relevant because the components of the force are close to zero.

**Table 4:** Experimental results obtained when testing the HP_1.a binder against 6 types of stone; arithmetic mean value of the results obtained for at least 12 output values (4 runs × 3 rounds) of V and F with the corresponding standard deviations (sd) and coefficients of variation (cv).

Type of Stone	V (g)	V (mm^3^)	cv	F (N)	cv
POR	0.04	5.53 ± 0.23	4.2%	16.00 ± 0.88	5.5%
	0.15	18.32 ± 0.95	5.2%	57.06 ± 2.57	4.5%
	0.28	34.65 ± 1.25	3.6%	109.42 ± 5.03	4.6%
RM	0.05	6.81 ± 0.31	4.5%	15.13 ± 0.64	4.2%
	0.14	17.82 ± 0.86	4.8%	41.31 ± 2.27	5.5%
	0.24	29.46 ± 1.71	5.8%	99.62 ± 6.08	6.1%
	0.43	52.72 ± 3.64	6.9%	155.72 ± 9.03	5.8%
AA	0.05	5.69 ± 0.39	6.8%	3.96 ± 0.15	3.9%
	0.19	23.27 ± 1.51	6.5%	47.59 ± 1.81	3.8%
	0.33	40.97 ± 1.84	4.5%	86.03 ± 4.65	5.4%
	0.45	55.69 ± 1.73	3.1%	129.00 ± 2.97	2.3%
CP	0.03	4.21 ± 0.22	5.2%	3.25 ± 0.19	5.7%
	0.14	17.08 ± 1.38	8.1%	24.04 ± 1.39	5.8%
	0.26	31.99 ± 2.82	8.8%	49.90 ± 2.54	5.1%
	0.42	52.17 ± 3.91	7.5%	120.28 ± 3.13	2.6%
SAB	0.05	5.94 ± 0.49	8.2%	17.23 ± 0.72	4.2%
	0.08	10.02 ± 0.79	7.9%	25.64 ± 0.92	3.6%
	0.19	24.09 ± 2.12	8.8%	57.35 ± 2.18	3.8%
	0.25	30.57 ± 2.11	6.9%	112.13 ± 5.49	4.9%
ME	0.01	1.07 ± 0.05	4.5%	15.96 ± 0.78	4.9%
	0.01	1.65 ± 0.10	6.3%	49.76 ± 2.94	5.9%
	0.03	3.71 ± 0.18	4.8%	160.94 ± 8.21	5.1%

## Data Availability

Data sharing is not applicable to this article.
